# Comprehensive response to Usutu virus following first isolation in blood donors in the Friuli Venezia Giulia region of Italy: Development of recombinant NS1-based serology and sensitivity to antiviral drugs

**DOI:** 10.1371/journal.pntd.0008156

**Published:** 2020-03-30

**Authors:** Ilaria Caracciolo, Erick Mora-Cardenas, Chiara Aloise, Tea Carletti, Ludovica Segat, Maria Sole Burali, Alexsia Chiarvesio, Vivianna Totis, Tatjana Avšič–Županc, Eloise Mastrangelo, Giuseppe Manfroni, Pierlanfranco D’Agaro, Alessandro Marcello

**Affiliations:** 1 Regional Reference Centre for Arbovirus Infections, Department of Medical, Surgical and Health Sciences, University of Trieste, Trieste, Italy; 2 Laboratory of Molecular Virology, International Centre for Genetic Engineering and Biotechnology (ICGEB), Padriciano, Trieste, Italy; 3 Azienda Sanitaria Universitaria Integrata di Trieste, UCO Igiene e Sanità Pubblica, Trieste, Italy; 4 Dipartimento di Scienze Farmaceutiche, Università degli Studi di Perugia, Perugia, Italy; 5 Centro Unico Regionale Produzione Emocomponenti C.U.R.P.E. P.O. Palmanova A.A.S.2 Bassa Friulana Isontina, Palmanova, Italy; 6 Laboratory of Diagnostics of Zoonoses and WHO Centre, Institute of Microbiology and Immunology, University of Ljubljana, Ljubljana, Slovenia; 7 CNR-Biophysics Institute, University of Milan, Milano, Italy; Fort Collins, UNITED STATES

## Abstract

Surveillance of Usutu virus is crucial to prevent future outbreaks both in Europe and in other countries currently naïve to the infection, such as the Americas. This goal remains difficult to achieve, notably because of the lack of large-scale cohort studies and the absence of commercially available diagnostic reagents for USUV.

This work started with the first identification of USUV in a blood donor in the Friuli Venezia Giulia (FVG) Region in Northern-Eastern Italy, which is endemic for West Nile virus. Considering that only one IgG ELISA is commercially available, but none for IgM, a novel NS1 antigen based IgG/M ELISA has been developed. This assay tested successfully for the detection of Usutu virus in blood donors with the identification of a second case of transmission and high levels of exposure. Furthermore, two pan-flavivirus antiviral drugs, that we previously characterized to be inhibitors of other flavivirus infectivity, were successfully tested for inhibition of Usutu virus with inhibitory concentrations in the low micromolar range.

To conclude, this work identifies North-Eastern Italy as endemic for Usutu virus with implications for the screening of transfusion blood. A novel NS1-based ELISA test has been implemented for the detection of IgM/G that will be of importance as a tool for the diagnosis and surveillance of Usutu virus infection. Finally, Usutu virus is shown to be sensitive to a class of promising pan-flavivirus drugs.

## Author summary

Tropical viruses transmitted by ticks or mosquitoes are becoming a health threat in areas of the world that were previously naïve to these infections. Usutu virus is a mosquito-borne virus that is circulating in Europe causing massive outbreaks in birds. Transmission to humans is documented, with some reports of severe neurological disease. However, the real size of transmission to humans suffers from lack of data due to insufficient surveillance. The first confirmed case of human USUV infection in an asymptomatic blood donor from North-Eastern Italy is hereby demonstrated by molecular assays and virus isolation. Specific Usutu virus serology has also been developed taking advantage of the NS1 viral antigen, which is tested on a number of blood donors demonstrating a high level of Usutu positivity. These findings confirm the human transmission in the region and offer a novel tool for specific Usutu virus surveillance. Finally, two drugs that were previously shown to have a wide spectrum of activity towards members of this family of viruses are shown to inhibit also Usutu virus, opening the way to a novel class antivirals.

## Introduction

Usutu virus (USUV), an arbovirus of the genus Flavivirus, family *Flaviviridae*, is an emerging virus in Europe that is gaining considerable attention [1]. USUV belongs to the Japanese encephalitis virus (JEV) antigenic complex, similar to West Nile virus (WNV), which is often circulating in the same areas. Similar to other flaviviruses, USUV is an enveloped virus with a (+)-strand RNA genome encoding a single polyprotein that is subsequently cleaved into structural (C, prM and E) and non-structural (NS1, NS2A, NS2B, NS3, NS4A, NS4B and NS5) proteins. USUV was initially isolated in Africa in 1959 and it has been continuously circulating with several introductions in Europe through migratory birds since the first documented cases in birds in Tuscany in 1996 and Vienna in 2001 [2–5]. Therefore, several distinct lineages of USUV (Europe 1–5 lineages) currently co-circulate [6]. Recently, a report of USUV infection of *Anatidae* in Belgium pose a risk of economically relevant zoonoses [7]. USUV is transmitted by different species of *Culex* mosquitoes, particularly *Culex pipiens*, which is thought to be the main vector in Europe [8]. However, the invasive mosquito *Aedes albopictus* is also capable of USUV transmission, albeit at a lower competence, and USUV was found naturally also in the invasive mosquito species *Aedes japonicus* [9, 10].

Humans, as well as other mammals, are dead-end hosts for USUV and show generally mild symptoms. However, neurological complications in immune-compromised patients could represent a growing concern for human health. Since 2009, several human cases of USUV-related neuroinvasive illness were reported in Italy [11–13], and in 2013, three other human cases were reported in Croatia [14]. USUV has also been recently associated with a clinical diagnosis of idiopathic facial paralysis in France [15]. Transfusion-transmitted USUV infection has not been reported so far. However, USUV-infected donations in the EU blood supply have been detected during routine screening of blood donations for WNV RNA [16–19]. USUV antibodies in blood donors had also been detected [20, 21]. The prevalence of USUV among blood donors is not fully determined also because there is no requirement to screen blood donors for USUV. Assessing the risk of USUV transmission through blood transfusion is therefore crucial. The cross-reactivity of WNV nucleic-acid tests (NAT) with USUV can contribute to the detection of these flaviviruses in donated blood [17, 18]. However, WNV NAT–reactive donations should undergo virus-specific confirmatory tests [22].

The identification and isolation of Usutu virus from a blood donor in the Friuli Venezia Giulia Region of North-Eastern Italy is hereby reported. Partial sequencing of the isolated virus mapped to the Europe 1 lineage. Importantly, an in-house IgG/M ELISA assay based on the NS1 antigen was therefore optimized in order to assess the seroprevalence of Usutu virus in this area. In addition, two novel pyridobenzothiazolone derivatives were also shown to be inhibitory against Usutu virus.

## Results

### Identification and isolation of USUV

**Fig 1:**
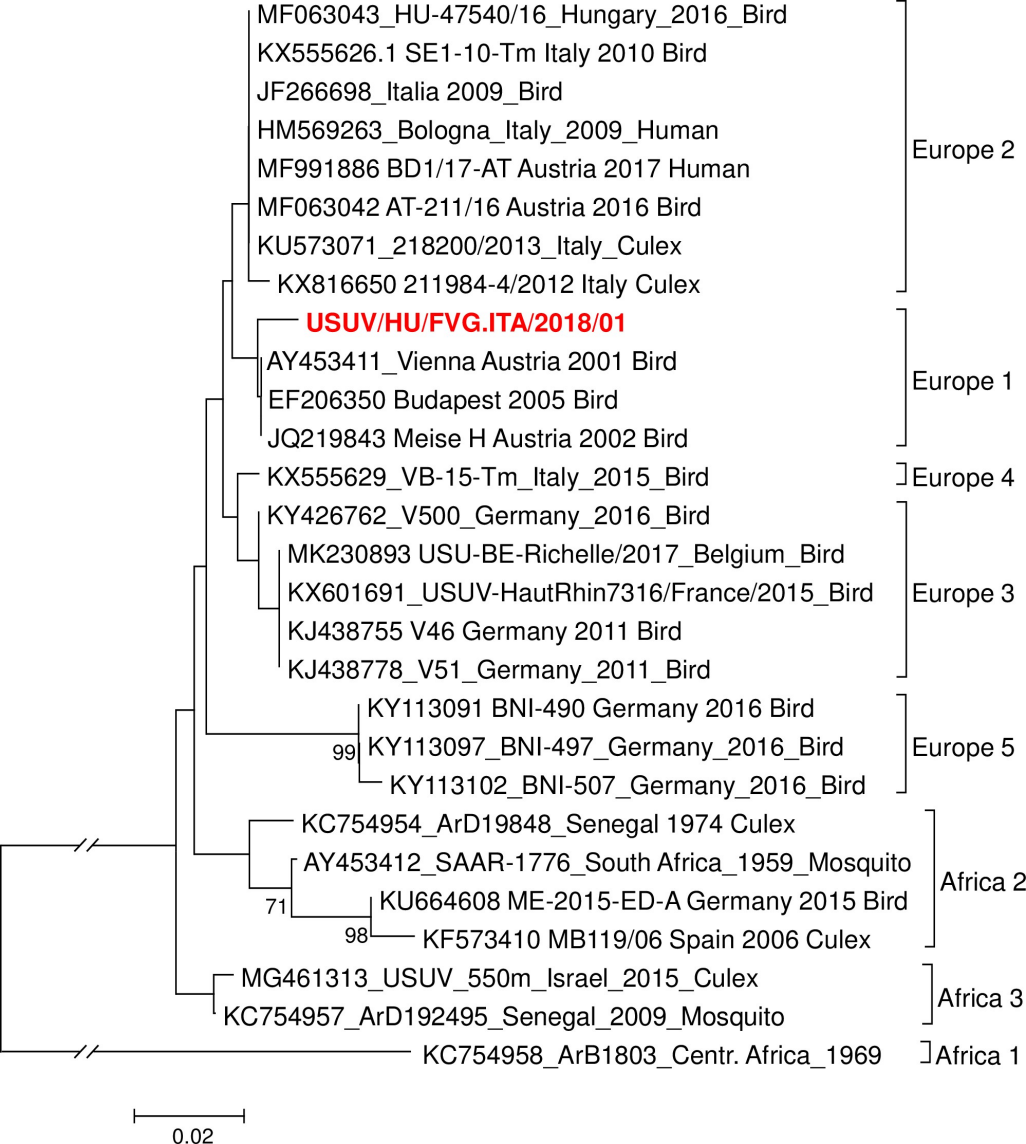
Phylogenetic analysis of USUV/HU/FVG.ITA/2018/01. Neighbor-Joining evolutionary history calculated with MEGA X software from curated USUV sequences following BLAST analysis and alignment of a fragment of 659bp within NS5. The evolutionary distances were computed using the number of differences method [58].

Blood donations in the FVG Region, an area endemic for WNV, are routinely screened by the Cobas WNV nucleic acid test and positive results are sent for confirmation to the Regional Reference Laboratory for Arbovirus infection in Trieste. In August 2018 an asymptomatic blood donor positive in the initial screening test could not be confirmed for WNV RNA. However, the sample turned positive for USUV RNA by the amplification protocol of Cavrini et al. [23]. The infectious virus was rescued from Vero cells inoculated with the human serum and a sequence of a 659 bp conserved region of NS5 was obtained (accession number MN509808). This novel isolate, named USUV/HU/FVG.ITA/2018/01 was shown to cluster with USUV strains from lineage Europe 01 isolated in Austria, Hungary and Germany (Fig 1A). This is the first documented case of USUV transmission to humans in Friuli Venezia Giulia (FVG) Region and is indicative of USUV circulation in the area.

### Development of a rNS1-based ELISA for USUV

**Fig 2:**
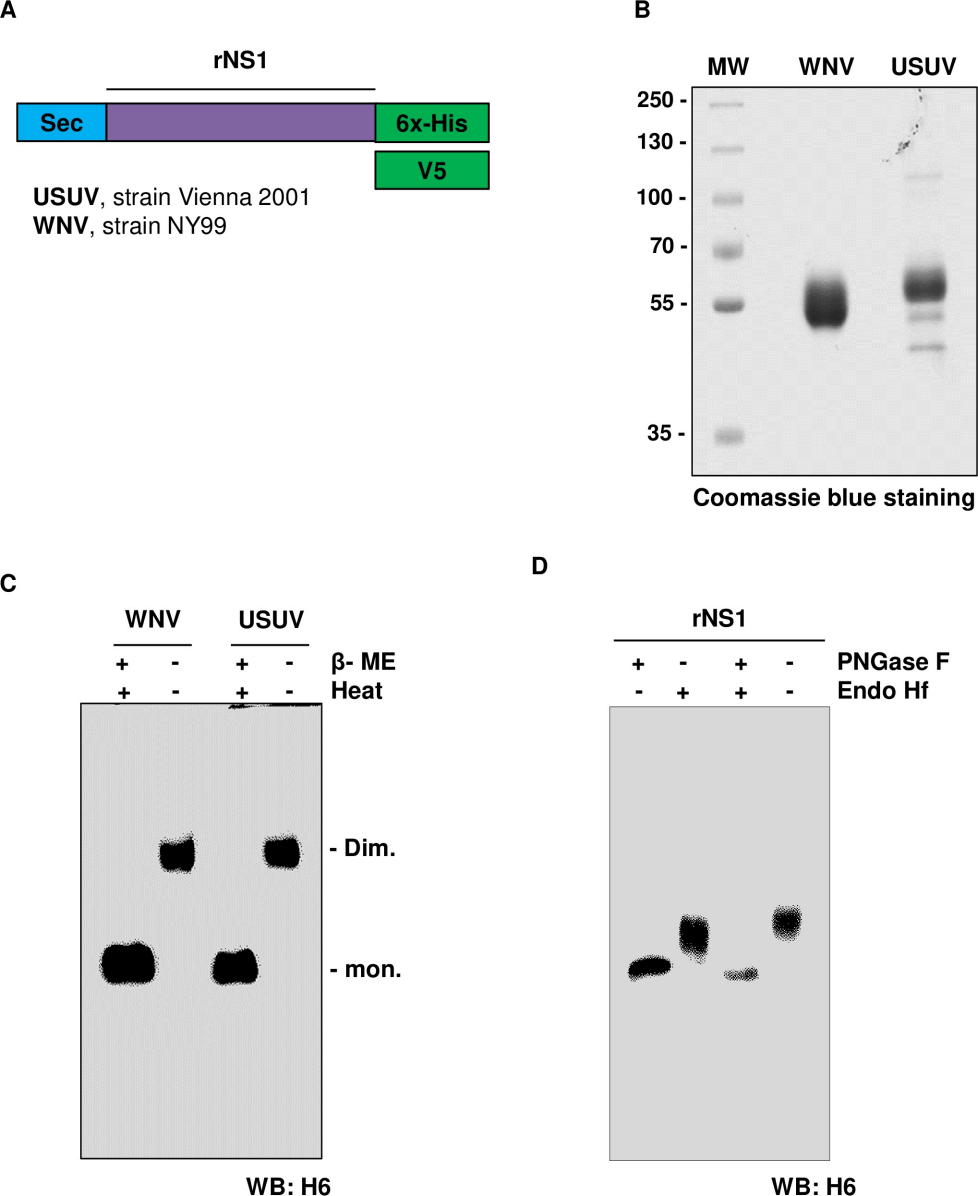
Purification of USUV rNS1. A) Schematic representation of expression constructs pcDNA3.1-sec-NS1-6xHis (upper) and pcDNA3.1-sec-NS1-V5 (lower) of both WNV and USUV expression constructs. Sec corresponds to an immunoglobulin leader sequence at the N-term; rNS1, recombinant nonstructural protein 1; 6xHis and V5 are tags cloned at the C-term for protein purification and mice immunization, respectively. Reference strains for USUV and WNV are also indicated. B) Coomassie blue SDS PAGE of purified rNS1 proteins: 2 μg of purified USUV and WNV rNS1 proteins were loaded as indicated. C) Western blot of purified USUV and WNV rNS1 in denaturing/reducing (line 1 and 3) and non-denaturing/non-reducing conditions (no heating and Loading Buffer without 2-βME) (line 2 and 4). rNS1 monomers (mon) and dimers (dim) are indicated (antibody against the His tag). D) Endoglycosidase analysis of purified USUV rNS1. USUV rNS1 was treated either with PNGase F or Endo Hf enzymes for 1.5 h at 37°C. WB analysis was assessed by 10% SDS-PAGE under standard conditions using an anti-6xHis-tag mAb (antibody against the His tag).

In order to study the serology of USUV, an NS1-based ELISA was established following a strategy described in detail elsewhere [24]. Expression plasmids encoding codon-optimized full-length nonstructural protein 1 (NS1) from USUV and WNV were generated. These constructs included a Sec leader peptide at the N-terminus of the protein for optimal secretion and a 6x-histidine tag (6x-His) or V5 tag at the C terminus for protein purification or mice immunization, respectively (Fig 2A). Secreted recombinant USUV NS1 (rNS1) was affinity purified by nickel chromatography from human embryonic kidney 293T (HEK293T) cells transiently transfected with these constructs. A control WNV rNS1 was also purified and both proteins were analyzed in parallel by SDS PAGE (Fig 2B). Thick bands around 55 kDa in size corresponding to the glycosylated forms of the protein were observed as expected. Densitometric analysis of the gel showed purity in the range of 87–90%, similar to commercially available NS1 proteins. To assess the native oligomeric state of the secreted proteins the purified rNS1 were analyzed by western blot under denaturing/reducing and non-denaturing/non-reducing conditions (Fig 2C). A band corresponding to the monomeric form of NS1 was observed when the sample was boiled and prepared in reducing Laemmli sample buffer, while only dimeric forms of NS1 proteins were observed when the western blot was performed in non-denaturing/non-reducing conditions. The glycosylation profile of purified rNS1 proteins was assessed by digestion with endoglycosidase Hf (Endo Hf) or peptide N-glycosidase F (PNGase F). After treatment for 1.5 hours at 37°C with Endo Hf, PNGase F or both enzymes, rNS1 proteins showed a shift in migration compared to untreated, as expected from a glycosylated protein (Fig 2D).

To assess the ability of rNS1 antigen to detect specific antibodies, Balb/c mice were immunized intradermal using a gene gun approach with an expression plasmid for USUV rNS1 carrying the V5 tag. Groups of four mice (Rx, Lx, RxLx, and unmarked) were immunized with the V5-tagged USUV rNS1 construct at fourteen days intervals and sera were collected at days 0 (pre-immune), 6, 22, 34 and 57 after the first immunization. The rNS1-based ELISA was used for IgM/G detection. The results are presented as a positive to negative (P/N) ratio calculated dividing the OD_450_ of test specimen by the OD_450_ of the negative control (mean of pre-immune sera). Cut-off values of P/N ratios were calculated based on the comparative receiver operating characteristic (ROC) curve analysis. The optimal cutoff values for IgM and IgG detection fell at 1.2 and 3 for both WNV and USUV, respectively. Each cut-off was selected based on the P/N ratio value that gave 100% sensitivity and specificity.

**Fig 3:**
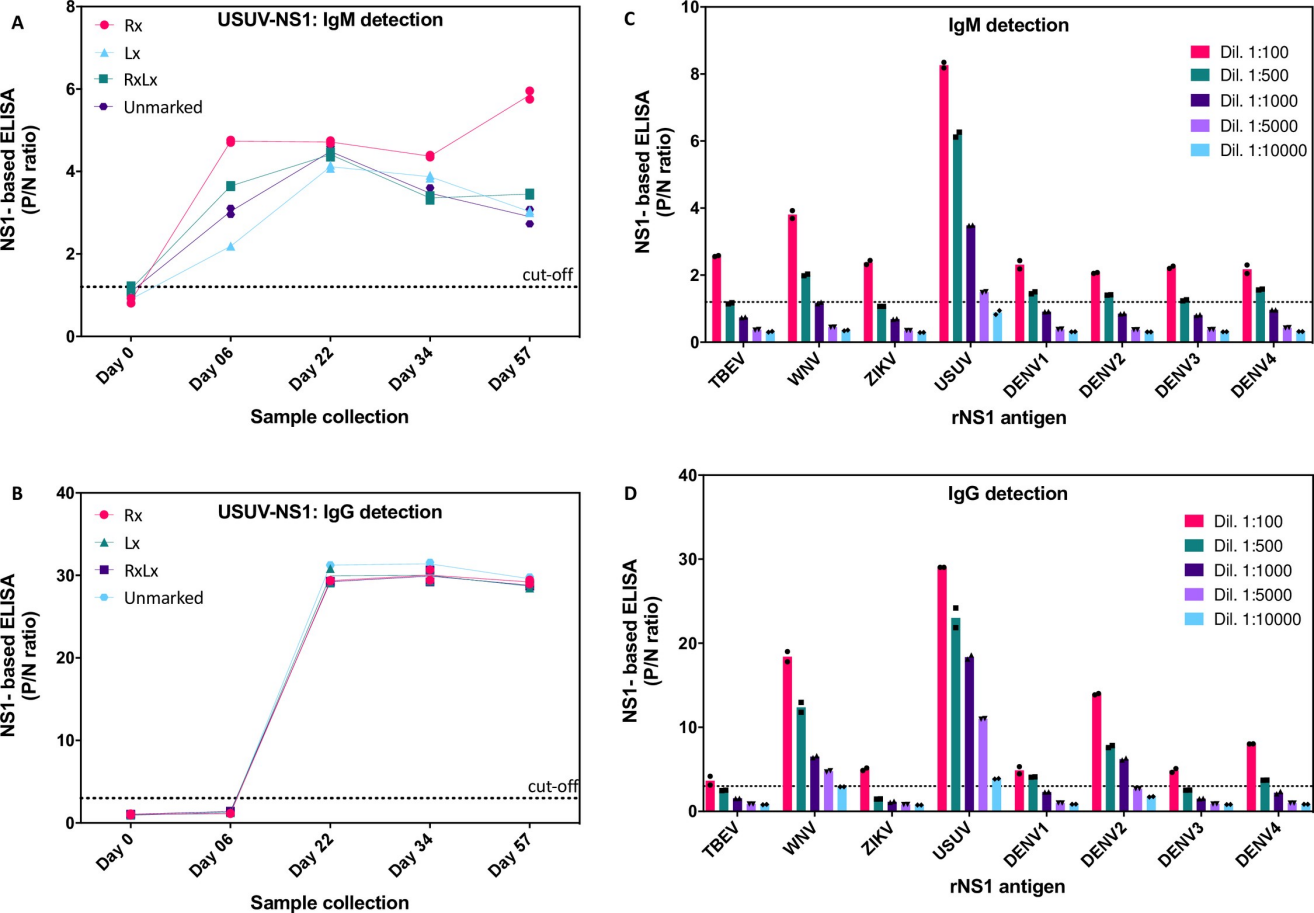
USUV rNS1-based ELISA in mice. A and B) USUV rNS1 IgM/G ELISA with sera from four different mice immunized with USUV rNS1 (mice were differentiated with a mark in the right ear (Rx), left (Lx), both ears (RxLx) or unmarked). Detection of IgM/G antibodies was performed at day 0, 6, 22, 34 and 57. C and D) TBEV, WNV, ZIKV, USUV and DENV1-4 IgM/G rNS1 ELISA with sera from USUV-NS1 immunized mice. Sera from day 57 post-immunization were diluted in PBS as indicated. Results are shown as the average of two biological replicates, single values are indicated by symbols on the graphs. Cutoff threshold is indicated by the dotted line.

Mice showed a robust immune response to USUV rNS1 with IgM detected as early as 5 days after the first immunization, followed by IgG (Fig 3A and 3B). Sera collected after the last bleeding was specific for USUV NS1 with some cross-reactivity with WNV, particularly for IgG, but lower cross-reactivity for TBEV, ZIKV or DENV1-4 (Fig 3C and 3D). A similar approach was used to test sera from mice immunized for WNV NS1 as described earlier [24]. As sown in S1A and S1B Fig, the WNV NS1 antigen is very specific for IgM/G from mice immunized with WNV NS1 compared to USUV NS1 and other flaviviruses. These data indicate that the WNV NS1 based ELISA is very specific for WNV versus USUV and other flaviviruses tested.

### Serology of USUV in blood donors

**Fig 4:**
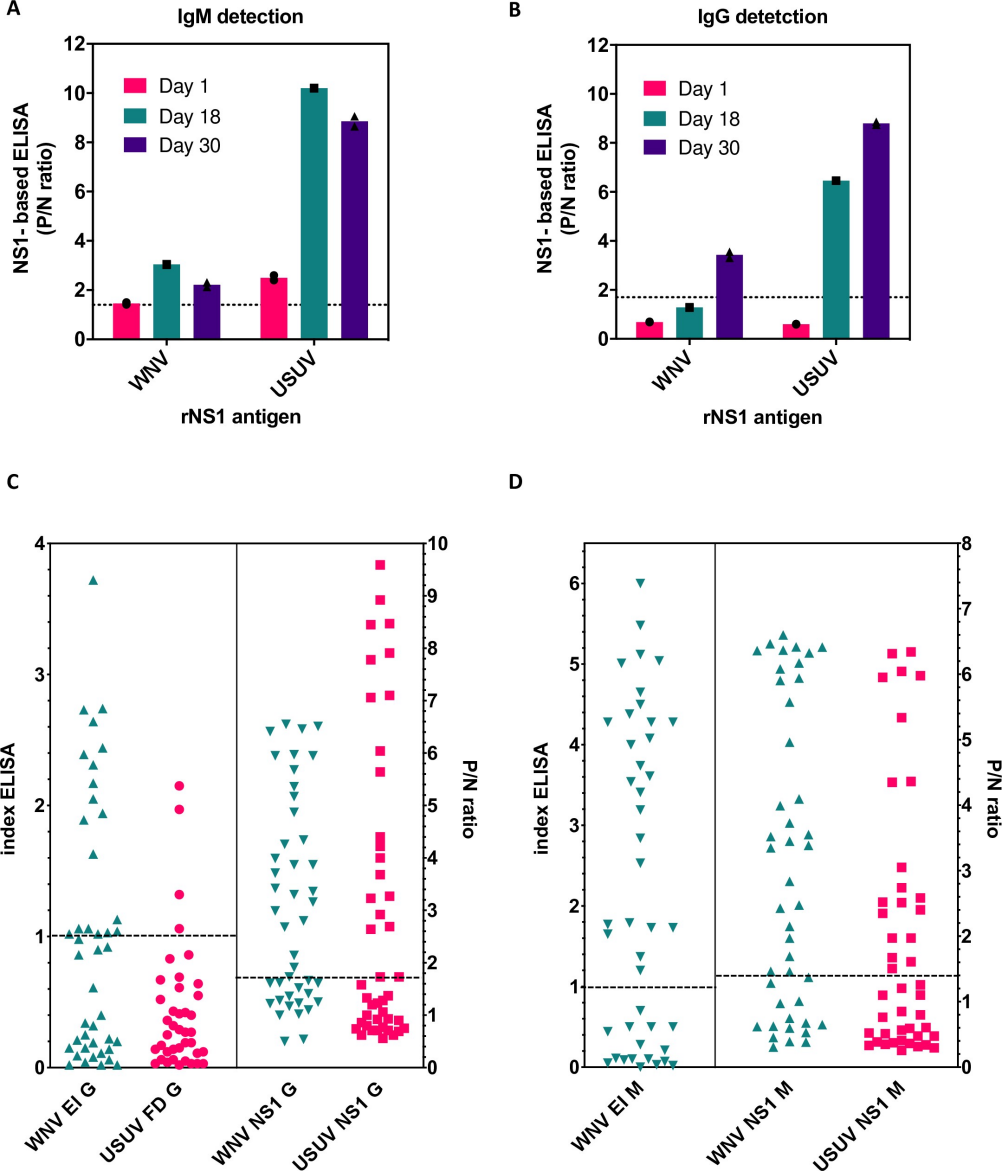
USUV rNS1-based ELISA in human samples. A and B) Three longitudinal sera samples from a single asymptomatic blood donor were tested at the indicated times for the presence of IgM (A) and IgG (B) antibodies against rNS1 antigen of USUV and WNV. C and D) Detection of IgG (C) or IgM (D) antibodies from 44 sera samples of blood donors by rNS1-based ELISA assay (EI and FD for commercial Eurimmun and Focus Diagnostics, respectively). Optimal cut-off value of WNV P/N ratio for IgG and IgM fell at 1.7 and 1.4, respectively, and was extended also to USUV for the analysis. Each ELISA result includes the average of two biological replicates. Results are shown as the average of two biological replicates, single values are indicated by symbols on the graphs. Cutoff threshold as determined in the text is indicated by the dotted line.

USUV rNS1-based ELISA for IgM/G detection was tested on follow up sera from the initial donor (1, 18 and 30 days from blood donation). WNV rNS1 was included because of the observed cross-reactivity in mice sera. As shown in Fig 4A and 4B, these sera showed USUV IgM/G seroconversion characteristic of a new infection, with low level of cross-reactivity for WNV observed for IgG responses. USUV seroconversion was also confirmed by plaque reduction neutralization test (PRNT_50_>10^2^).

A total of 44 sera samples from blood donors that showed positive in the Cobas WNV nucleic acid test in the summer of 2018 were then chosen to detect IgG antibodies to USUV or WNV by the novel NS1-based ELISA established in this work. 10/44 samples confirmed WNV new infections by RT PCR and additional 13 by IgM ELISA (Eurimmune AG, Luebeck), while none for USUV. These samples were tested for the detection of IgG antibodies to WNV by the Focus Diagnostics commercial ELISA assay (plates coated with recombinant WNV prM/E antigen [25] and by the commercial Euroimmun USUV IgG ELISA assay (plates coated with purified USUV E protein).

High seroprevalence of IgG antibodies to WNV was observed (Fig 4C), with 93% (41/44) and 25% (11/44) of the total number of samples that were IgG positive by rNS1-based ELISA or commercial ELISA assays, respectively. A high percentage of IgG positive sera samples to USUV was also detected with the in-house assay (Fig 4C), with 48% (21/44) of positive samples, while only 9% (4/44) were positive for USUV using the commercial Euroimmun IgG ELISA assay. The cutoff established for WNV (1.7) was used also for USUV since a sufficient number of certified USUV samples was not available for ROC analysis. To note 21/44 samples were positive for WNV and negative for USUV in the rNS1 assay, 20/44 WNV+/USUV+, 2/44 WNV-/USUV- and 1/44 WNV-/USUV+. The latter donor was tested again 10 days after the first sampling and confirmed negative for WNV and positive for USUV with a P/N IgG ratio growing from 3.2 to 4.88. This patient was also tested for NS1 IgM confirming seroconversion for USUV with P/N IgM ratio growing from 6.6 to 10.6, while remaining negative for WNV IgM. PRNT neutralization data confirmed USUV infection (PRNT_50_>10^2^).

Next all 44 sera were also tested for WNV IgM using both Eurimmun WNV IgM ELISA and with rNS1 ELISA, while for USUV only rNS1 ELISA could be performed given the lack of a commercial assay for the purpose. Positive WNV IgM sera were detected (Fig 4D) in 63% (27/43) and 66% (29/44) using the commercial or rNS1 ELISA, respectively, while USUV rNS1 ELISA showed 45% (20/44) IgM positive samples using the cutoff of 1.4 established for WNV. 11/44 sera were WNV+/USUV-, 18/44 WNV+/USUV+, 13 WNV-/USUV- and 2 WNV-/USUV+ including the sample tested above. The 18/44 sera that showed double positivity were further examined singularly. As shown in S1C Fig, 8/44 samples were significantly positive for both WNV and USUV. 7/44 samples showed high WNV values and borderline USUV values. Considering that the threshold for USUV IgM ELISA could not be established precisely these samples may be considered WNV+. Conversely 3/44 sera showed the opposite pattern and could be considered USUV+. One sample showed borderline values for both and could be considered negative.

These data show that an additional donor could be confirmed as newly USUV infected by rNS1-based ELISA and the establishment of a novel IgM ELISA for USUV detection. However, further analysis on human sera from exposed individuals will be necessary to complete the evaluation of this novel assay.

### Pyridobenzothiazolones activity towards USUV

**Fig 5:**
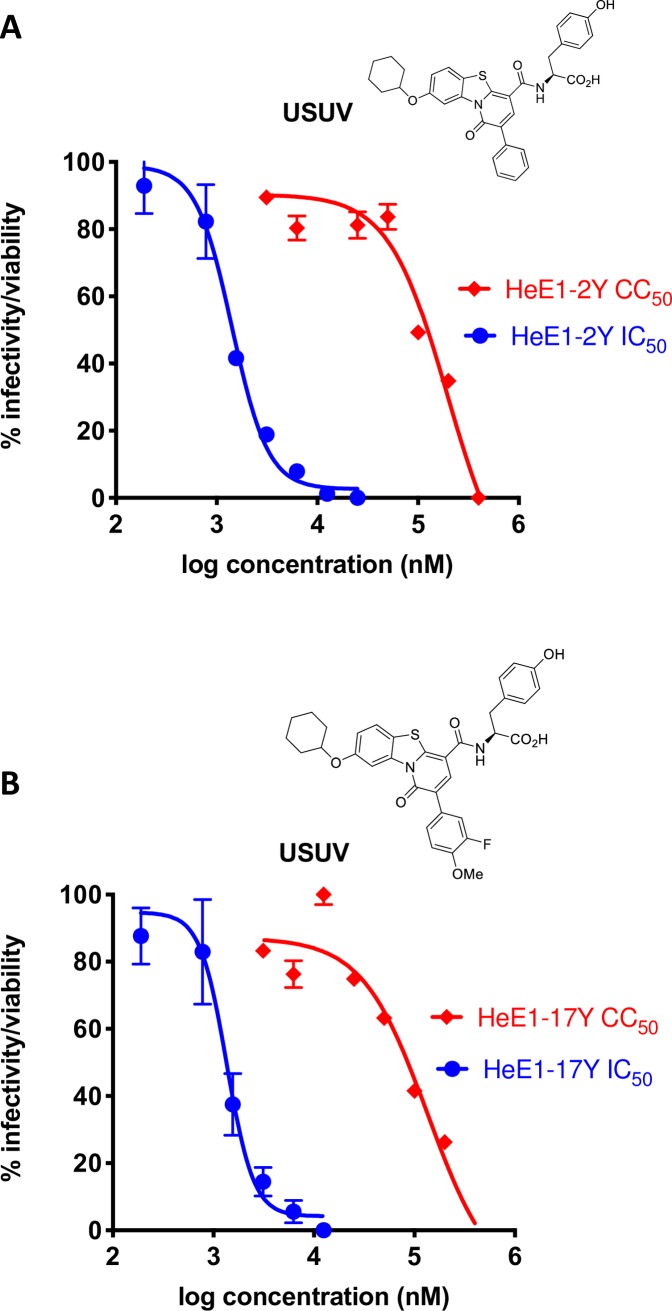
Activity of Pyridobenzothiazolone derivatives against USUV. (A and B) Pyridobenzothiazolone derivatives HeE1-2Y (panel A) and HeE1-17Y (panel B) were tested for antiviral activity against USUV by the plaque reduction method in Vero cells (blue line). In parallel, cytotoxicity was measured by the Alamar blue method (red line).

USUV is quickly becoming a relevant infection in Europe and may spread worldwide. Therefore, it is important to initiate antiviral testing as soon as possible. Pyridobenzothiazolone derivatives have been shown to be potent pan-flavivirus inhibitors that block viral infectivity [26, 27]. In particular the derivatives HeE1-2Y and HeE1-17Y (Fig 5) have a half inhibitory concentration (IC_50_) between 0,5–10 μM against a range of Flavivirus with a good selective index. As shown in Fig 4, HeE1-2Y and HeE1-17Y showed an IC_50_ for USUV in Vero cells of 1.4±0.4 μM and 1.3±0.3 μM, respectively, with a half-cytotoxic concentration (CC_50_) >100 μM in both cases. The result indicates that USUV inhibition is in the range of other flaviviruses tested previously [27].

## Discussion

The FVG region of Italy is endemic for flaviviruses with reported transmission to humans such as tick-borne encephalitis virus (TBEV) and WNV [28] (National surveillance and response integrated plan for West Nile and Usutu viruses—2018. Rome: Italian Ministry of Health; 2019. Available from: http://www.trovanorme.salute.gov.it/norme/listaAtti?page=1). Therefore, blood donors are routinely screened for WNV infection. USUV co-circulates with WNV in many European countries and share host and vector species [13]. The year 2018 showed a dramatic increase of USUV and WNV infections in Europe with increased transmission to humans [18, 29], including the neighboring Veneto Region [30]. Furthermore, circulation of USUV in mosquitoes has been described in the region [31]. However, in this report for the first time, the presence in FVG of USUV transmission to humans is being documented by isolation and a retrospective serological analysis. Two samples of serum from 44 blood donors were confirmed as new infections with USUV, one also by NAT and virus isolation. Genetic analysis indicates that the isolate belongs to the Europe 1 lineage circulating in Italy and neighboring countries at least since 2009 [5].

Given the increased incidence of USUV, it is critical to develop specific serological assays for diagnosis and surveillance. However, only the Eurimmune USUV IgG ELISA is commercially available, while for IgM there are none on the market. The Eurimmune USUV IgG ELISA is based on the viral structural proteins and these assays suffer from broad antigenic cross-reactivity of anti-flavivirus antibodies [32]. To this end the use of secreted flavivirus NS1 as an antigen alternative to virion proteins for the detection of IgM/G has proven to provide a better level of specificity [24, 33–38]. NS1 is secreted as a glycosylated oligomer in high amounts during the viremic phase and its detection is considered a marker of infection [39]. Anti-NS1 IgM appear early (2–3 days) following infection. Evaluation of acute infection by measuring anti-NS1 IgM antibodies has shown higher sensitivity compared to RT-PCR in sera samples of infected patients with dengue virus [40, 41]. Detection of IgG antibodies using purified NS1 antigens from different flaviviruses has been reported to show a low degree of cross-reactivity to related viruses and high reactivity to homologous NS1 antigens [24, 33]. Available commercial assays for USUV are scarce and exploit mostly virion components as antigen. In this report, IgM/G ELISA based on recombinant NS1 for USUV is being established and tested in comparison with the highly homologous and geographically overlapping WNV. Careful analysis in immunized mice allowed a better characterization of sensitivity and specificity showing that immune IgM sera for TBEV, WNV, ZIKV, and DENV1-4 didn't cross-react with USUV, while some cross-reactivity for WNV, and to a lesser extent to DENV2 persisted for IgG detection. Retrospective serological analysis in a restricted group of blood donors with suspected WNV infection confirmed a second USUV infection and overall 45% (20/44) USUV IgM positive sera, with at least 7/44 that were also positive for WNV IgM. Although cross-reactivity cannot be completely ruled out, the latter samples may also indicate co-infections with WNV and USUV occurring in the same patient. This hypothesis is sustained by the fact that viruses co-circulate in the same area and share hosts, vectors and seasonality. Furthermore, recent report identified human cases with co-infection by WNV and USUV in the same individual [18, 42, 43].

In this report, it has also been shown that HeE1-2Y and HeE1-17Y compounds are active against USUV in the lower micromolar range. These data add to the so-far scarce armamentarium of antiviral compounds tested for their activity against USUV, and flavivirus in general [44]. Indeed, only few reports investigated antiviral drugs against USUV. In addition to interferons, which were shown to be active against USUV [45], other compounds include: Ribavirin (RBV), favipiravir (FAV), 5-fluorouracil (FU) and 2’-C-methyl nucleoside analogs such as 2’-Cmethyladenosine (2’CMA) that elicit antiviral activity towards USUV at 10–100 uM with a low selective index [46, 47], Hypolipidemic drugs targeting the Acetyl Coenzyme A Carboxylase such as the 5-(tetradecyloxy)-2-furoic acid (TOFA) or 3,3,14,14-tetramethylhexadecanedioic acid MEDICA 16 [48] or the autophagy inhibitors 3-methyladenine and wortmanin that significantly reduced USUV replication in Vero cells [49]. Although HeE1-2Y and HeE1-17Y compounds were tested only for one USUV strain, it is expected that they will be active also for other circulating strains because of the little genetic variation among isolates observed so far [50, 51]. However, further analysis will be required to follow up on these data, particularly concerning mechanism of action and optimization.

In conclusion, the first documented cases of USUV transmission to humans in the Regione FVG have been identified in this work. A better survey of the population and of the vector will be required to establish if the area is endemic for the virus. To this end, the development of a novel NS1-based ELISA test not only allows the study of the high seroprevalence of USUV infection in blood donors of the Region, but also provides a tool for further epidemiological and clinical use. Finally, USUV is shown to be sensitive to the action of Pyridobenzothiazolone derivatives, a novel class of promising pan-flavivirus compounds.

## Materials and methods

### Identification of USUV

The first screening of blood donors was routinely performed with the Cobas WNV nucleic acid test (Roche). Confirmation of USUV RNA was obtained as described earlier [23]. The primers (USU-F 5’-AAAAATGTACGCGGATGACACA-3’, USU-R 5’-TTTGGCCTCGTTGTCAAGATC-3’) amplified a partial sequence (73 bp) of USUV NS5 gene, which was detected by a dual-labeled probe (USU-P 5’-6famCGGCTGGGACACCCGGATAACC-tamra-3’).

Sequence analysis was performed on a PCR amplification product of 659bps from the NS5 gene using primers USU-9170F and USU-9704R [52] and MAMD and cFD [53]. The evolutionary history was inferred using MEGA X software [54, 55]. The phylogenetic tree was obtained by the Neighbor-Joining method on amino acid sequences; the Kimura two-parameter method was used to calculate nucleotide substitutions, and a bootstrap of 500 replicates lead to evaluate significance of tree topology. Sequence data were directly submitted to GenBank with accession number: BankIt2264908 USUV/HU/FVG.ITA/2018/01, MN509808.

### Cloning, expression, and purification of rNS1

Cloning strategy was similar to what has been described previously for WNV NS1 [24]. Briefly, the nucleotide sequences coding for USUV NS1 was derived from the NCBI database entry of USUV strain Vienna 2001 (AY453411). Sequence was codon-optimized for the expression in *Mus musculus*, synthesized commercially by *Gene Art Gene Synthesis* (Thermo Fisher Scientific) and sub-cloned into the pcDNA3.1 expression vector (Life Technologies) fused to an immunoglobulin leader sequence (Sec) at the N-terminus. The genes were kept in-frame with the polyhistidine (6x-His) tag or the V5 tag at the C-terminus. Transient transfection of HEK293T (source ATCC CRL-7216) cells was performed by the standard calcium phosphate method. 16h after transfection, cells were washed twice in phosphate-buffered saline (PBS) and further cultured for 30h in serum-free media supplemented with 5 mM of sodium butyrate to increase expression of rNS1. Culture supernatants were then cleared by centrifugation at 4000 g for 4 min and purified by affinity chromatography on FPLC using HiTrap Chelating HP 5mL columns (GE Healthcare). Eluted rNS1 in Imidazole was concentrated and buffer-exchanged to PBS using Ultra-4 centrifugal filters devices (Amicon, 10K). The purity and concentration of the purified protein were estimated by Coomassie blue staining and by Bradford assay, respectively. Immunoblots were performed using the monoclonal antibody anti-6x-His-tag (Sigma). Signals were visualized by ECL (Luminata Crescendo, Merck Millipore). WNV, DENV, TBEV and ZIKV rNS1 proteins were purified with a similar protocol as described elsewhere.

For the glycosylation analysis, purified NS1 proteins were digested for 1.5 hours with endoglycosidase Hf (Endo Hf) or/and Peptide-N-Glycosidase-F (PNGase) endoglycosidases according to manufacturer's protocols (New England Biolabs).

### Mice immunization

Four 5–6 weeks old, female Balb/c mice per each condition were immunized intradermally with a plasmid encoding for rNS1 by Gene Gun technology (Bio-Rad, Hercules, CA, USA). A scheme based on prime-boost immunization was followed. Before the immunization, the abdominal area of each mouse was shaved and 1 μm gold particles coated with 1 μg of plasmid DNA (V5-tagged constructs of rNS1 USUV) were delivered by biolistic particle system at 400 psi. Each group of mice was immunized 4 times with a specific plasmid at fourteen days intervals. Blood samples were collected by sub-mandibular puncture before immunization (pre-immune sera) and 5–7 days after each boost (bleeding I, II, III and IV). Sera samples were collected and stored at -20°C until use.

### Clinical samples and ethics statement

All clinical samples has been collected and analyzed by the Regional reference Centre for Arbovirus infections as part of the “Piano nazionale integrato di sorveglianza e risposta ai virus West Nile e Usutu– 2018” of the Italian Ministry of Health.

Animal care and treatment were conducted in conformity with institutional guidelines after approval by the ICGEB Institutional Review Board following consent from the Italian Ministry of Health in accordance with the Italian law (D.lgs. 26/2014), following European Union policies (European and Economic Council Directive 86/609, OJL 358, December 12, 1987).

### NS1-based Enzyme-Linked Immunosorbent Assay (rNS1-based ELISA)

Nunc Maxi Sorp Immuno-Plates (ThermoFisher-Nunc, Roskilde, Denmark) were coated with 5 μg/ml of purified rNS1 antigens in 50 mM Na_2_CO_3_/NaHCO_3_ buffer pH 9.6 and incubated overnight at 4°C. Plates were washed with Phosphate-Buffered Saline (PBS) buffer and blocked with 2% milk in PBS for 45 min at room temperature (RT). After washing, 100 μl of 1:100 sera dilutions (or serial dilutions when it is indicated) of sera from immunized mice or 1:20 dilutions of sera from infected humans were added to each well. For IgM detection, sera samples were pre-adsorbed with GullSorb (Meridien Bioscience) and incubated for 1 hour at 37°C, while for IgG detection sera samples were incubated for 1 hour at RT. 100 μl/well of HRP-linked goat antibodies anti-mouse IgM/G or anti-human IgM/G were used (Sigma, 1:5000). In all cases, secondary antibodies were incubated for 1 hour at RT. After each antibody incubation, wells were washed three times with PBS 0.1% tween 20 (PBST). Signal was developed by adding 70 μl of 3,3',5,5'-Tetramethylbenzidine (TMB) substrate (Sigma). The reaction was stopped by adding 30 μl of 2N H_2_SO_4_ to each well. The optical density was measured at 450 nm (OD_450_) with an ELISA EnVision 2104 Multilabel Plate Reader (Perkin Elmer). The P/N ratio for both IgM/IgG rNS1-based ELISA was obtained by dividing the OD_450_ of test specimen by the mean OD_450_ of 16 negative control specimens. Commercial ELISA Eurimmun USUV IgG EI 2667–9601 G and Focus DxSelect WNV IgG EL0300G were used for USUV and WNV IgG detection, respectively. The comparative receiver operating characteristic (ROC) curve analysis was used to calculate the optimal cut-off values of P/N ratios for IgM/G detection by WNV rNS1-based ELISA. A bin range of different P/N ratio values was used to select the optimal cut-off value that gave 100% sensitivity and specificity [24].

### Plaque reduction neutralization assay (PRNT)

PRNT was performed on Vero cells as previously described [56, 57]. Briefly, Heat-inactivated (56°C for 30 minutes) serum samples were serially diluted two-fold in DMEM, mixed with equal volumes of WNV or USUV (50 PFU) and incubated for 1 hour at 37°C. Vero cells seeded on 24 wells’ plates were incubated with the mixture for 1h at 37°C and then overlaid with 3% carboxymethylcellulose (CMC, Sigma) in DMEM 2% FBS. After 3 days, cells were fixed 3.7% paraformaldehyde and stained with 1% crystal violet for 30 min. Plaques were counted manually. The neutralization titer (PRNT_50_) was calculated as the reciprocal of the highest test serum dilution for which the virus infectivity was reduced by 50% when compared with the average plaque count of the challenge virus.

### Pyridobenzothiazolone derivatives

HeE1-2Y and HeE1-17Y were re-synthesized for this study following the chemical procedures previously reported [26, 27]. Vero E6 cells were infected with USUV (MOI = 1) in the presence of different concentrations of compound HeE1-2Y and HeE1-17Y [27]. The cell supernatant was collected and titrated 24 h.p.i. on Vero cells by the plaque assay to measure infectivity. IC_50_ and CC_50_ values were estimated from triplicate independent experiments using GraphPad (non-linear fit, log inhibitor vs normalized response).

## Supporting information

S1 Fig A and B) TBEV, WNV, ZIKV, USUV and DENV1-4 IgM/IgG rNS1 ELISA with sera from WNV-NS1 immunized mice. C) Detection of IgM antibodies from 18/44 WNV+/USUV+ sera samples of blood donors tested by the USUV rNS1-based ELISA assay. Results are shown as the average of two biological replicates. Cutoff threshold is indicated by the dotted line and determined as described in the text.
(TIF)
