# Supplementary figures and images for: Comprehensive response to Usutu virus following first isolation in blood donors in the Friuli Venezia Giulia region of Italy: Development of recombinant NS1-based serology and sensitivity to antiviral drugs
Source: PLoS Negl Trop Dis. 2020 Mar 30;14(3):e0008156. doi: 10.1371/journal.pntd.0008156 (PMC7145266; doi:10.1371/journal.pntd.0008156)

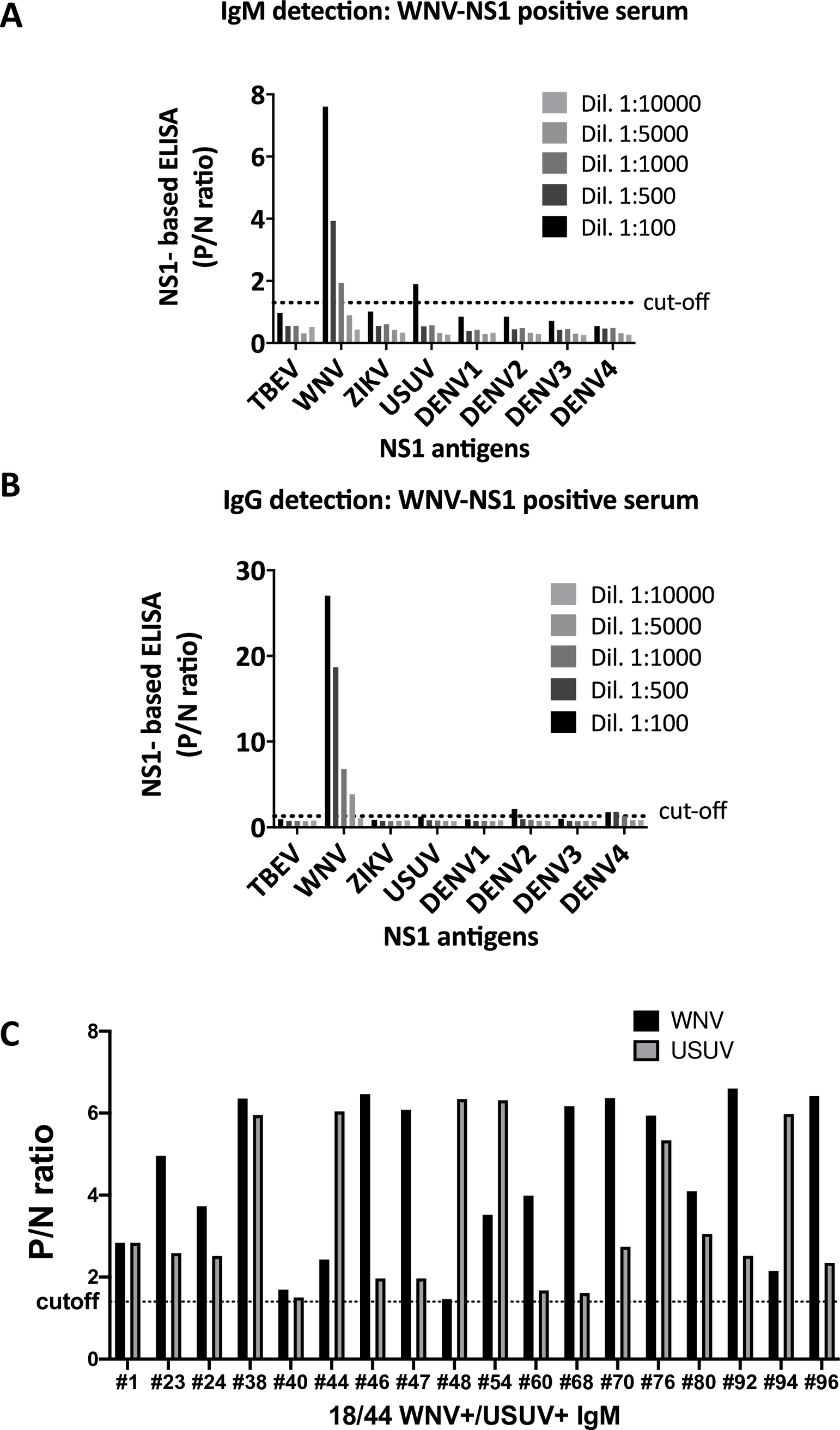

Supplement: S1 Fig — A and B) TBEV, WNV, ZIKV, USUV and DENV1-4 IgM/IgG rNS1 ELISA with sera from WNV-NS1 immunized mice. C) Detection of IgM antibodies from 18/44 WNV+/USUV+ sera samples of blood donors tested by the USUV rNS1-based ELISA assay. Results are shown as the average of two biological replicates. Cutoff threshold is indicated by the dotted line and determined as described in the text. (TIF) [file pntd.0008156.s001.tif]
